# Efficacy of a polyvalent mastitis vaccine against *Staphylococcus aureus* on a dairy Mediterranean buffalo farm: results of two clinical field trials

**DOI:** 10.1186/s12917-017-0944-4

**Published:** 2017-01-19

**Authors:** Jacopo Guccione, Antonella Pesce, Massimo Pascale, Caterina Salzano, Gianni Tedeschi, Luigi D’Andrea, Angela De Rosa, Paolo Ciaramella

**Affiliations:** 1 Department of Veterinary Medicine and Animal Productions, University of Napoli “Federico II”, Via Delpino 1, 80137 Naples, Italy; 2 Istituto Zooprofilattico del Mezzogiorno, Via A. Jervolino 19, 81100 Tuoro Caserta District, Italy; 3 Veterinary practitioner, Caserta District, 81100 Italy; 4 Hipra Italia s.r.l., Via Franciacorta 74, 25038 Rovato, Italy

**Keywords:** *Staphylococcus aureus*, Vaccination, Prophylaxis, *Bubalus bubalis*, Udder health

## Abstract

**Background:**

In the last years the knowledges on Mediterranean Buffalo (MB) mastitis is remarkably improving, nevertheless the attention has been never focused on vaccination as preventive strategy for the control of mastitis in these ruminates. The aim of the current study was to assess clinical efficacy over time of two different preventive vaccination protocols against *S. aureus* mastitis, in primiparous MB.Vaccinated (VG) and not-vaccinated (N-VG) groups, of 30 MB each one, were selected from two different herds (herd A: VG1 and N-VG1; herd B: VG2 and N-VG2) of the same farm. Herd A received a double vaccination (Startvac®, 45 and 10 days before calving, protocol A), while in herd B an additional administration was performed (52 days after calving, protocol B). Bacteriological milk culture and assessment of somatic cell count (SCC) were performed at 10, 30, 60 and 90 days in milk (DIM) from composite milk samples. After 90 DIM, daily milk yields and SCC values were monthly detected until dry-off.

**Results:**

The overall incidence of positive MB for *S. aureus* was 40.8% (49/120) in VG1 and 43.3% (52/120) in N-VG1 (Protocol A), while 45.8% (55/120) and 50.8% (61/120) in VG2 and N-VG2 (Protocol B). The latter was associated with a significant decreased in prevalence (at 90 DIM) and incidence of mastitis (animals positive for *S. aureus*, SCC > 200^10^3^, with or without clinical signs) in the vaccinated MB, while no difference occurred in protocol A. Moreover, herd B showed a significant reduction in prevalence of intramammary infection (animals positive for *S. aureus,* SCC < 200^10^3^, no clinical signs) in the vaccinated MB at 60 DIM while no differences were detected in herd A, at any sampling time; N-VG2 had significantly higher overall SCC values than VG2 (4.97 ± 4.75 and 4.84 ± 4.60 Log10 cells/mL ± standard deviation, respectively), while no differences were recorded in herd A.

**Conclusions:**

The current investigation explores for the first time the clinical efficacy of vaccinations against *S. aureus* infections in MB, showing encouraging results regarding reduction in mastitis and somatic cell count; the polyvalent mastitis vaccine may be considered an additional tool for in-herd *S aureus* infection and should be associated to other control procedures to maximize its properties.

## Background

Mastitis represents one of the major causes of economic loss in dairy Mediterranean buffaloes (MB) because negatively influencing milk quality and yields of the animals affected [1, 2]. In MB mastitis is mainly caused by several contagious (e.g. *Staphylococcus aureus*, *Streptococcus agalactiae*), environmental (e.g. *Streptococcus uberis* and *Streptococcus dysgalactiae*, *Escherichia coli*, *Enterobacteriaceae*, and yeasts), and opportunistic (e.g. coagulase-negative staphylococci) bacteria [3–5].

*S. aureus* is one of the most important pathogens, causing clinical and subclinical mastitis in dairy MB and cows all over the world [2, 6, 7]; Clinical outcomes and high within-herd prevalence were recently described in MB confirming its relevance as contagious microorganism [2]. This bacteria typically colonizes the injured skin; damage of the teat end and faulty milking encourages migration of bacteria into the udder causing intramammary infections (IMI) and sometimes persisting for extended periods [4, 8, 9]. Some strains are particularly resistant to antibiotics [4] and moderate results were reported in an our previous study exploring the effects of an antibiotic selective treatment [1]. As a consequence, similarly to dairy cows additional strategies of herd health management including treatment or culling of affected animals, implementation of biosecurity measures and hygienic milking practices, have been recommended to prevent and control udder health problems related to *S. aureus* in MB farms [2].

Although with different outcomes, great scientific attention was recently given to mastitis control by means of preventive vaccination protocols in cows [10–16]. A commercial multivalent vaccine containing inactivated *S. aureus* and *E. coli* has been available in European Union for the last years and several investigations regarding its usefulness were recently performed in cows with different results [13, 14, 17]. In MB, the awareness for mastitis are remarkably improving, although the attention has been never focused before on vaccination as preventive strategy for mastitis.

Considering the premises, the aims of the present study was to evaluate the clinical efficacy of a polyvalent commercial vaccine administered in dairy primiparous MB evaluating as outcomes (1) the prevalence and incidence of IMI and mastitis, (2) the effects on somatic cell count and (3) milk yield.

## Methods

### Animals and Farm Management

All the animals chosen in the present study were reared in the same breeding farm of approximately 700 dairy buffaloes, free from mandatory reportable diseases and located in Caserta area (Southern Italy). The eligible criteria for the farm were represented by 3, monthly and consecutive samplings of bulk tank milk positive for *S. aureus* before the beginning of the study, analyzed by means of PCR-based assay as described by Syring et al. [18]. No strict criteria were instead applied for bulk milk somatic cell count (SCC) or mastitis incidence. Differences observed about herd management practices were recorded during the period of the investigation (two consecutive years) to exclude possible influences on vaccination efficacy.

Farm was characterized by herringbone parlor and animals were milked twice a day. A mean of 247 ± 23-day milk yield per head of 2200 kg while mean bulk tank SCC values of 172 × 10^3^ ± 49 × 10^3^ and 179 × 10^3^ ± 32 × 10^3^ cell/mL were recorded for the whole herd during the first and the second year of investigation, respectively. All MB selected were kept in a roofed common paddock of ~ 1200 m^2^ (~30 m × 40 m) As often observed in MB’s farms, before parturition and until 40–50 DIM, all the *primiparous* MB were housed separated from the *pluriparous*. Milking was performed in the same milking parlor, although the *primiparous* animals were milked first. After 50 DIM, *primiparous* were mixed with the *pluriparous* animals, including milking time. The barn was characterized by a common bedded area (with dried manure solids), a loafing area and a feeding alley with solid non-grooved concrete floor (cleaned twice a day). The entire herd was fed a total mixed ratio including hay, silage and a multi-vitamin integrator three times a day; free access to the protected water trough was always guaranteed.

### Study design

Two herds (A and B) were enrolled in the current study. Sixty *primiparous* for each herd were subdivided into two groups of 30 MB: vaccinated and not-vaccinated (herd A: VG1 and N-VG1; herd B: VG2 and N-VG2). Animals were chosen from the same farm in two consecutive lactations (herd A: I year, December 2012 to November 2013 - herd B: II year, December 2013 to November 2014). Every year the animals were chosen by means convenience sampling from 80 *primiparous* in good health having 4 functional quarters, teats free of significant lesions, and with an estimated calving date to allow vaccination at predicted times before calving. Samples size was calculated by using the formula proposed by Thrusfield [19] considering the following values: study population (pregnant primiparous MB in farm, ~14% of entire herd – approximately stable parameter in both years considered), expected prevalence of positive primiparous MB for *S. aureus* (55%) [1], confidence interval 95% and desired absolute precision (5%).

All the animals were individually submitted to a complete clinical examination with particular focus on the udder health status following the clinical procedure described by Ciaramella [20].

For each herd the progressive vaccination was performed on 50 MB were chosen by means of simple randomization; out of the latter, the first 30 animals reaching the entire vaccination protocol were enrolled as VG. The other 30 not vaccinated subjects were selected as N-VG.

On sampling day, local and systemic signs and changes in milk appearance, such us overall appearance of depression, udder swelling and pain, off-color and watery appearance of milk, and presence of flakes, clots or pus, were recorded. California Mastitis Test (CMT) was routinely performed from each composite milk sample, with values ≥1 interpreted as positive [2] (data not shown).

### Vaccine

Commercial polyvalent mastitis vaccine (*STARTVAC*®, HIPRA Spain Co. Ltd, Girona) containing inactivated *E. coli* J5 and inactivated *S. aureus* (CP8) strains SP 140, expressing slime-associated antigenic complex (SAAC) was used [21].

The farmer was previously informed about the purposes and methods of the two clinical field-trials and the study received the approval by the Ethical Animal Care and Use Committee of University of Studies of Naples “Federico II”. During the present investigation each herd was submitted to a different vaccination protocol: the first (protocol A - herd A) was based on two intramuscular vaccine injections located at the medium third of the neck (2 mL each one) and performed at 45 and 10 days before the estimated date of calving on VG1; the second one (protocol B - herd B), instead included an additional administration on VG2 at 52 days in milk (DIM).

### Milk Samples

All milk samples were aseptically collected in sterile test tubes immediately before regular evening milking, as described by National Mastitis Council [22] for dairy cows. At 10, 30, 60 and 90 DIM, a sample from each quarter was collected; a composite milk sample was created by mixing equal amounts of milk (50 mL) from all 4 quarters into a sterile test tube (BD Vacutainer, Oxford, UK) and used to perform bacteriological milk culture (BMC) and determine the SCC; finally, milk yields were also recorded during the same samplings time using automatic dedicated software (Afifarm, Afimilk, Kibbutz Afikim, Israel). After 90 DIM, milk yield and SCC values were evaluated at 150 and 240 DIM (close to dry off). As described by a previous study [1], a great variability to achieve the supposed drying-off time (daily milk yield < 0.5 L/day) was detected in the enrolled animals (from 240 to 260 DIM), as consequence, the last sample considered useful for the statistical and economic analysis was collected at 240 DIM.

### Definition of Udder Health Status

Following the classification reported in previous studies [1, 23], the animals enrolled were defined as negative for *S. aureus,* affected by IMI or mastitis considering: presence/absence of clinical signs, SCC values, and microbiological status; briefly, MB producing milk with SCC below the threshold of 200 × 10^3^ cells/mL and associated with BMC negative to *S. aureus* were considered and hereafter referred to as “healthy udder”; animals with analogous SCC values and positive BMC caused by *S. aureus* have been instead considered affected by IMI; moreover, buffaloes producing milk with SCC upon the threshold of 200 × 10^3^ and positive BMC for *S. aureus* have been defined as affected by mastitis, with (clinical mastitis - CM) or without clinical signs (subclinical mastitis - SCM) [1, 23]. All the MB affected by mastitis (CM and/or SCM) due to *S. aureus* were considered in end-point phase and excluded from the study.

### Cytological and bacteriological investigations

Composite milk samples were placed in a cool box (4 °C) and brought to the reference laboratory within 1 h (h) of collection, where they were submitted to SCC analysis and BMC within 2 h of collection. SCC was determined using an automatic analyzer, approved for buffalo milk (Fossomatic 5000, Foss Electric, Hillerod, Denmark) [24]. The BMC and bacteriological identification were performed according to guidelines of the National Mastitis Council [25]. Briefly, 10 μL of each milk sample was streaked on one quarter of a blood-agar plate (Merck KGaA, Darmstadt, Germany), incubated at 37 °C for up to 48 h and examined two time (at 24 and 48 h of incubation). Bacterial colonies were tentatively identified on gross morphology; number and types of colonies were also recorded. As described in previous studies [2], when 3 or more dissimilar colony types were isolated on the plate, the sample was considered contaminated. Gram staining and catalase testing were performed to differentiate between streptococci and staphylococci, and tube coagulase testing using rabbit plasma to differentiate between coagulase-positive and coagulase-negative staphylococci. A final identification of microorganisms was performed using the colorimetric automated identification system (Vitek 2 XL 120; bioMerieux Inc., Hazelwood, MO), according to the manufacturer’s instructions. Enterobacteriaceae were grown on MacConkey agar (Oxoid, Basingstoke, UK) and identified using the same automated system. Isolates identified with confidence levels greater than 0.90 were considered identified mastitis pathogens at species level; otherwise, they were identified at genus level.). All the laboratory procedures were performed without previous informations about the results of the clinical examination of the udder (physical and CMT).

### Statistical analysis

Different animal health status, SCC, and milk yields were analyzed by standard descriptive statistics and normality was assessed using histograms, normal probability plots and Shapiro Wilk tests. Data were expressed as absolute numbers, percentage, or mean ± SD. Log- transformation was applied to variable not normally distributed. Untransformed and log-transformed continuous variables (SCC and milk yields) were compared using Student’s *t*-test. Significant differences between expected and observed frequencies of categorical data (i.e. vaccine efficacy: VG1 vs. N-VG1 and VG2 vs. N-VG2) were assessed by means of contingency tables, using *χ* ^2^-test. Fisher’s exact test was performed in case of low expected frequencies (<5). *P*robabilities <0.05 were considered significant. Moreover, a mixed models were also used to investigate the direct effects of the vaccination intra- and inter-protocols. The final model included fixed time effect, fixed group effect, fixed interaction time*group and buffalo as random effect. For statistical analysis of the outcomes a mixed linear models was used for continuous data (SCC and milk yield); Bonferroni correction was used to set *P*-value for multiple statistical test; the threshold for statistical significance in our analysis was *P =* 0.0052, the null hypothesis was rejected for *P* <0.01.

For categorical data (presence/absence of mastitis) a mixed logistic models based only on fixed group effects and buffalo-specific random effect was performed because otherwise the function to be maximized was not concave due to the small amount of observations. Data inter-protocols (absolute values) were combined in the model including time and group as fixed effects. *P*robabilities <0.05 were considered significant. All the statistical data were analyzed using dedicated software (STATA 12.0, StataCorp LP, Collage Station, Texas, USA).

## Results

### Course of the study and prevalence of udder pathogens

No general or local adverse reactions to the use of the vaccine was observed in all the buffaloes of both the protocols after the administrations. The request number of vaccinated animals was reached in 1.5 months in herd A and in 2 months in herd B. Vaccinations were performed 45.8 ± 4.1 and 11.1 ± 2.4 days pre-calving in VG1, while 46.2 ± 4.6 and 10.3 ± 2.2 days-pre calving in VG2; the additional vaccination expected for protocol B was instead administered 52.2 ± 1.1 days post-calving. During the two years of study, no differences about all the aspects of herd management practices (e.g. feeding and housing system, milking routine, dairy workers, therapeutic protocols for mastitis, etc.) were detected.

Throughout the study, 480 composite milk samples were collected and submitted to BMC. All udder pathogens isolated during the first 90 DIM in vaccinated and not-vaccinated groups of the two herds and their relative incidence are reported in Table 1. Briefly, the most commonly isolated pathogens in both the groups of the two herds, were *S. aureus* followed by coagulase-negative staphylococci. Positive-culture status for *S. aureus* was detected in 30.2% (49/162) and 32.5% (52/160) of the overall bacteriological results in VG1 and N-VG1, respectively; it was instead recorded in 41.9% (55/131) and 50.4% (61/121) of the samples in VG2 and N-VG2, respectively. *E. coli* was only detected in both groups of herd B (VG2: 1 time, 0.8% and N-VG2: 2 times, 1.7%, Table 1) and associated with not severe CM (mild milk change, CMT ≥2).

**Table 1:** Bacteriological results of all samples collected in both the herds of *primiparous* Mediterranean buffalo during the first 90 days in milk of investigation. Percentages represent the proportion calculate on the total number bacteriological results

	Herd A				Herd B			
	VG1		N-VG1		VG2		N-VG2	
Pathogens	No.	Percent	No.	Percent	No.	Percent	No.	Percent
*Staphylococcus aureus*	49	30.2	52	32.5	55	41.9	61	50.4
*Coagulase-negative staphylococci*	32	19.9	32	19.3	41	31.3	16	13.1
*Enterococcus faecalis*	2	1.2	2	1.3	1	0.8	1	0.8
*Aerococcus viridans*	4	2.5	4	2.5	2	1.5	4	3.3
*Streptococcus dysgalactiae*	2	1.2	1	0.6	0	0	2	1.7
*Streptococcus thoraltensis*	2	1.2	2	1.3	0	0	1	0.8
Other gram-positive pathogens (not *S. aureus*)	2	1.2	5	3.1	3	2.3	2	1.7
*Escherichia coli*	0	0	0	0	1	0.8	2	1.7
*Acinetobacter lwoffii*	*4*	2.5	3	1.9	0	0	2	1.7
Bacillus spp.	0	0	0	0	0	0	1	0.8
Culture negative	65	40.1	59	36.9	28	21.4	29	24.0
Total	162	100	160	100	131	100	121	100

No = number; VG1 and N-VG1 = Vaccinate Group1 and Not-Vaccinated Group1, respectively; VG2 and N-VG2 = Vaccinate Group1 and Not-Vaccinated Group2, respectively. Herd A: received two doses of vaccine pre-calving; Herd B: received two doses of vaccine pre-calving and one post-calving

The overall percentage of positive samples for other udder pathogens, except for *S. aureus* and *E. coli,* was 29.6% (48/162) in VG1 and 30.6% (49/160) in N-VG1, while it was 35.9% (47/131) and 23.9% (29/121) in VG2 and N-VG2, respectively.

Negative-culture status was observed in 40.1% (65/162) and 36.9% (59/160) of the all bacteriological results observed in VG1 and N-VG1, respectively; 21.4% (28/131) and 24.0% (29/121) were instead recorded in VG2 and N-VG2, respectively.

### Vaccine effects

#### Protocol A

The 40.8% (49/120) and 43.3% (52/120) of the samples collected were positive for *S. aureus* in VG1 and N-VG1. No bacteria were detected at 10 DIM (0/30) in VG1, while their presence increased up to 16.7% (5/30), 60.0% (18/30) and 89.2% (25/28) at 30, 60 and 90 DIM, respectively; in N-VG1 the prevalence of positive-cultures for *S. aureus* was instead of 3.3% (1/30), 33.3% (10/30), 53.6% (15/28) and 92.8% (26/28) at 10, 30, 60 and 90 DIM, respectively; no statistically significant difference was found regarding this variable between VG1 and N-VG1 at any sampling time.

Details regarding of the effects of *S. aureus* on udder health are reported in Table 2. Briefly, two CM due to *S. aureus* were recorded both in VG1 (6.6%, 2/30) and N-VG1 (6.6%, 2/30), at 60 and 30 DIM, respectively. During the investigation, no SCM due to *S. aureus* were detected between 10 and 60 DIM in both groups, while they were observed both in VG1 (14.3%, 4/28) and N-VG1(10.7%,3/28) at 90 DIM. No statistically significant difference between prevalence of mastitis in VG1 and N-VG1 was observed. During the first 90 DIM, no statistically significant difference was recorded between the incidence of mastitic animals in VG1 (20.0%, 6/30) and N-VG2 (16.7%, 5/30). Regarding the prevalence of MB affected by IMI or with healthy udder, data are reported in detail in Table 2; no statistically significant difference between prevalence of IMI or healthy udder was found in VG1 and N-VG1 at any sampling time.

**Table 2:** Udder health status in primiparous Mediterranean buffalos within the two study herds (A and B) in association with *Staphylococcus aureus* detection in composite milk samples

Herd A									
		10 DIM		30 DIM		60 DIM		90 DIM	
Udder health status	Groups	No.	Percent	No.	Percent	No.	Percent	No.	Percent
CM	VG1	0/30	0	0/30	0	2/30	6.6	0/28	0
	N-VG1	0/30	0	2/30	6.6	0/28	0	0/28	0
SCM	VG1	0/30	0	0/30	0	0/30	0	4/28	14.3
	N-VG1	0/30	0	0/30	0	0/28	0	3/28	10.7
IMI	VG1	0/30	0	5/30	16.7	16/30	53.3	22/28	78.6
	N-VG1	1/30	3.3	8/30	26.6	15/28	53.6	23/28	82.1
Healthy	VG1	30/30	100	25/30	83.3	12/30	40.0	2/28	7.1
	N-VG1	29/30	96.7	20/30	66.7	13/28	46.4	2/28	7.1
Herd B									
CM	VG2	0/30	0	0/30	0	0/28	0	0/27	0
	N-VG2	0/30	0	0/27	0	0/23	0	0/20	0
SCM	VG2	0/30	0	2/30	6.6	1/28	3.6	3/27^a^	11.1
	N-VG2	3/30	10.0	4/27	14.8	3/23	13.0	5/20^b^	25.0
IMI	VG2	2/30	6.6	12/30	40.0	14/28^a^	50.0	21/27	77.8
	N-VG2	1/30	3.3	15/27	55.5	20/23^b^	86.9	13/20	65.0
Healthy	VG2	28/30	93.3	16/30	53.3	13/28^c^	19.1	3/27	11.1
	N-VG2	26/30	86.7	8/27	29.3	0/23^d^	0	2/20	10.0

VG1 and N-VG1 = Vaccinate Group1 and Not-Vaccinated Group1, respectively; VG2 and N-VG2 = Vaccinate Group1 and Not-Vaccinated Group2, respectively. CM = Clinical Mastitis; SCM = Subclinical Mastitis; IMI = Intramammary infection; DIM = Days in Milk. Herd A: received two doses of vaccine pre-calving; Herd B: received two doses of vaccine pre-calving and one post-calving. Animals affected by mastitis (SCM or CM) were considered in end- point. ^a,b^ *P* <0.01, ^c,d^ *P* <0.001

Details regarding of the effects on SCC and milk yield are reported in Table 3. Briefly, during the protocol A, the overall averages of Log_10_ SSC values observed during the whole lactation were 4.93 ± 4.71 Log_10_ cells/mL in VG1 and 4.95 ± 4.78 Log_10_ cells/mL in N-VG1 (IMI + healthy udder MB); no statistically significant difference was found between the two values as well as between the daily mean values of SCC recorded at all sampling times in VG1 and N-VG1. Regarding the milk yields, the overall mean values observed in the same animals (IMI + healthy udder MB) during the entire investigation were 9.70 ± 2.07 L/day in VG1 and 9.35 ± 1.57 L/day in N-VG1; no statistically significant difference was recorded for this parameter at any sampling time.

**Table 3:** Daily means somatic cell count values (Log_10_ cells/mL ± SD) and milk yields (Litres ± SD) detected in VGs and N-VGs in both the herds of *primiparous* Mediterranean buffalo during 240 days in milk of study

	DIM					
Item	10	30	60	90	150	240
Herd A -Log_10_ SCC						
VG1	4.84 (±4.51)	4.73 (±4.62)	4.82 (±4.54)	5.10 (±4.75)	5.02 (±4.90)	5.08 (±4.95)
N-VG1	4.88 (±4.65)	4.80 (±4.68)	4.87 (±4.77)	5.05 (±4.92)	4.98 (±4.89)	5.09 (±4.78)
Herd B -Log_10_ SCC						
VG2	4.86 (±4.60)^a^	4.67 (±4.45)^a^	4.86 (±4.71)	4.97 (±4.66)	4.84 (±4.51)^a^	4.85 (±4.67)^c^
N-VG2	4.99 (±4.74)^b^	4.94 (±5.01)^b^	4.87 (±4.69)	4.87 (±4.73)	4.98 (±4.67)^b^	5.15 (±4.64)^d^
Herd A –Milk Yields						
VG1	7.85 (±0.85)	13.10 (±2.56)	13.04 (±2.77)	12.19 (±2.59)	8.63 (±2.13)	3.37 (±1.51)
N-VG1	7.31 (±2.36)	12.89 (±2.20)	13.20 (±2.21)	11.63 (±1.48)	8.01 (±0.56)	3.05 (±0.59)
Herd B –Milk Yields						
VG2	8.50 (±2.04)	13.39 (±2.27)	13.66 (±2.15)	13.87 (±2.89)	8.84 (±1.94)	3.98 (±1.77)
N-VG2	7.7 (±2.12)	13.08 (±2.53)	13.10 (±2.83)	12.41 (±2.34)	9.57 (±4.89)	4.34 (±1.57)

VG1 and N-VG1 = Vaccinate Group1 and Not-Vaccinated Group1, respectively; VG2 and N-VG2 = Vaccinate Group1 and Not-Vaccinated Group2, respectively; DIM = days in milk; ^a,b^ *P* <0.05,^c,d^ *P* <0.01. Herd A: received two doses of vaccine pre-calving; Herd B: received two doses of vaccine pre-calving and one post-calving

#### Protocol B

The 45.8% (55/120) and 50.8% (61/120) of the samples cultured were positive for *S. aureus* in VG2 and N-VG2, respectively. The prevalence of positive-cultures for *S. aureus* in VG2 was 6.6% (2/30) at 10 DIM and increased up to 46.7% (14/30), 53.6% (15/28) and 88.9% (24/27) at 30, 60 and 90 DIM, respectively; in N-VG2 it was instead of 13.3% (4/30), 70.4% (19/27), 100% (23/23) and 90.0% (18/20) at 10, 30, 60 and 90 DIM, respectively; a statistically significant difference was found between VG2 and N-VG2 at 60 DIM (*P* < 0.001).

Details regarding of the effects of *S. aureus* on udder health are reported in detail in Table 2. No CM due to *S. aureus* were recorded during the entire protocol in both groups (VG2 and N-VG2). SCM were detected at 30, (6.6%, 2/30), 60 (3.6%,1/28) and 90 DIM (11.1%,3/27) in VG2, while in N-VG2, they were detected at each sampling time with a prevalence ranged between 10% and 25%. A statistically significant difference was found at 90 DIM (*P* <0.01). During the first 90 DIM, a statistically significant difference between the incidence of animals affected by mastitis in was also recorded (VG2: 20.0% - 6/30 and N-VG2: 50.0% - 15/30, *P <*0.05). According to the mixed logistic model the VG2 animals had less 23% of probability to develop mastitis compared to N-VG2 (*P* <0.001). Regarding the prevalence of MB affected by IMI and with healthy udder a statistically significant difference between prevalence of MB affected by IMI in VG2 and N-VG2 was found at 60 DIM (*P* <0.01), while between healthy udder at 60 DIM (*P* <0.001).

Details regarding of the effects on SCC and milk yield are reported in Table 3. Briefly, during the protocol B, the overall average of Log_10_ SSC values observed during the whole lactation were 4.84 ± 4.60 Log_10_ cells/mL in VG2 and 4.97 ± 4.75 Log_10_ cells/mL in N-VG2 (IMI + healthy udder MB); a statistically significant difference was found between the two values (*P* <0.05). A statistically significant difference was also recorded between daily mean values of SCC in VG2 and N-VG2 at 10 (*P* <0.05), 30 (*P* <0.05), 150 (*P* <0.05) and 240 DIM (*P* <0.01). Regarding the milk yields, the overall mean values observed in the same animals during the entire investigation were 10.37 ± 2.18 L/d in VG2 and 10.03 ± 2.28 L/d in N-VG2; no statistically significant difference was recorded at any sampling time between the two groups. The mixed linear model confirmed a significant difference between mean SCC of VG2 and N-VG2 at 150 (−66611.1, *P* <0.001) and 240 (−76444.4, *P* <0.01) DIM. No difference where instead detected regarding milk yields.

Finally, combining the two field trial, the mean difference of SCC between VG2 and N-VG2 was significantly higher than the analogous difference between VG1 and N-VG1 (−19778.06, *P* <0.05). No significant differences were instead observed regarding the milk yields.

## Discussion

For the purposes of the analysis outlined in this paper, the efficacy of two different regimens of vaccination, based on the use of a multivalent commercial inactivated bovine vaccine against *S. aureus* mastitis, was compared for the first time in MB during the lactation. Estimation of vaccine effects against contagious infections and mastitis under field conditions is an interesting challenge in species like MB where the knowledges regarding the preventions of mastitis are truly rare.

The high prevalence of mastitis due to *S. aureus* mono-infection reported in these ruminants [1], has been confirmed also in the present investigation. In this context, the influence of the dilution effects of *S. aureus* due to composite milk samples use has been not considered substantial, as described for cows [26]. Instead, the lack of quarter milk samples could represent a restriction of the present study because of lower sensibility of IMI detection and loss of useful information on quarter level (e.g. new infection rate, bacteriological cure rate, duration of the infections).

During the survey, only *primiparous* MB were enrolled because our previous studies showed a high-frequency isolation of *S. aureus* in first lactating animals between 30 and 90 DIM [1, 2, 5] and also to exclude the potential negative influence of a poor management of dry period. The complete lack of previous knowledge concerning preventive vaccination against mastitis in MB, lead the authors in a careful and methodical approach for the evaluation of the outcomes of a vaccine produced in origin for dairy cows; for this reason, two different regimens were carried out according to the manufacturer's instructions verifying the tolerance of the animals to the product, the effects on udder health, the milk quality and yields.

As described by Schukken et al. [13] for cows, also for MB the analysis of the data regarding the efficacy estimates was focused on the animals that received the full vaccination protocols; despite the ambition to vaccinate the animals according to the vaccination schemes planned, some difficulties were detected to achieve the necessary number of vaccine doses planned because of a continuous and precise scheduling required, inaccurate estimation of duration of pregnancy, early loss of pregnancy, abortions or MB calving pretermly. Given that incomplete vaccination due to the reasons described could be a common finding also in many MB herds, it is possible that our estimates of vaccine efficacy could be overestimated compared with the efficacy observed under true field conditions.

The importance of *S. aureus* as a contagious pathogen has been widely recognized both in dairy buffaloes and cows because cause of mastitis and relevant economic losses due to its negative influence on milk quality and yields [2, 6]. A previous study investigating clinical relevance and genotype of *S. aureus* in MB, elucidated that (1) genotype B was the only one detected, (2) it was associated to high within-herd prevalence (up to 55%) and incidence (up to 20.4%, during the whole lactation period), (3) it was responsible of contagious mastitis [2]. As well established also for cows, some strains of *S. aureus* (e.g. genotype B) are related to high contagiousness, causing herd problems with a within-herd prevalence of up to 87% [27]. A high prevalence and incidence of positive MB for *S. aureus* was also detected in both protocols and groups of the current investigation confirming the high-frequency isolation of this pathogen. Moreover, considering the overall cultured milk samples, *S. aureus* was also the most frequently isolated bacterium in both the herds and groups followed by coagulase-negative staphylococci. The result confirms a similar findings recently observed both in MB [2] and cows herds [28] where the percentage of isolation of coagulase-negative staphylococci and *Streptococci* were indeed considerably lower in herds positive for *S. aureus* showing high contagiousness and pathogenicity.

A significant difference in the overall rate of mastitis, comparing unvaccinated and vaccinated MB, was observed only in herd B. The administrations of 3 doses of vaccine, may significantly reduce the incidence of mastitis within the herd; the difference seems to be related to the lower prevalence of SCM detected at any sampling time in the VG2 than N-VG2 and achieving a significant peak at 90 DIM. Therefore, the novel contribution of the current study has been to show encouraging results regarding the efficacy of the inactivate vaccine on MB, in contrast with some previous similar studies on staphylococcal vaccination in dairy cows described relatively low vaccine efficacy or no vaccine efficacy at all [15, 17]. However, it is necessary to underline that another of the restrictions of the present investigation may be represented by the chosen study design: indeed only the direct vaccine effects, without attempt to estimate the overall population estimates, were described; as a consequence, it possible that our outcomes may be overestimated if compared with the truly observed vaccine efficacy under overall population conditions. Furthermore, the presence of CM in herd A (VG and CG) suggests that the protocol based on 2 vaccine administrations had not effect to prevent the appearance of clinical signs; on the contrary, in herd B, no difference has been found in both the groups considered overtime and therefore it has been not possible to evaluate this effect. The finding merits further scientific attention, because of the small number of animals enrolled and the lack of previous studies performed on MB useful to compare the results observed.

Regarding IMI due to *S. aureus,* a high prevalence of MB infected was recorded in both the protocols although lower rate of infection was observed in VGs than N-VGs in almost all the samplings. During the investigation a higher prevalence of infections was always observed between 60 and 90 DIM in both the protocols and groups; these effects may have been caused by mixing *primiparous* with *pluriparous* animals in the herds, as initiated by the farmer between 40 and 50 DIM. It is interesting to highlight how the administration of the third dose seems to decrease significantly the prevalence of IMI between VG2 and N-VG2 at 60 DIM, conversely to the use of two doses. Moreover, the moderate reduction of IMI observed in this sampling time, may have consequently reduced the mastitis prevalence observed in the successive control time; the finding described may be also supported by the significant higher percentages of healthy udder MB recorded in VG2 than N-VG2 at 60 DIM. The results observed seem less encouraging than those recently described for cows where vaccination resulted in moderate reduction in incidence of new infections and more pronounced reduction in duration although in presence of an efficacy for transmission still relatively low [13]. As reported by several studies in cows [27–29], also in our investigation the vaccine effects on IMI may be influenced by farm-specific characteristics, such as strain types and farm management practices, and not be only ascribed to the vaccine’s properties. Excellent preventive herd management strategies may contribute to significantly reduce the overall herd exposure to *S. aureus* both in MB and cows [2, 13, 30], resulting in an important contribution to indirect vaccine efficacy.

Regarding the effects of vaccination on milk yield and quality, no significant differences were observed between the overall mean milk yields and daily mean production between the two groups of both protocols, while a significant difference between the two regimens was observed regarding SCC. As reported in Table 3, the protocol A was not able to positively influence SCC values in VG1 compared with N-VG1, differently from the protocol B, in which the additional vaccine’s dose may have produced an additional immunization of VG able to reduce *S. aureus* multiplication in the mammary gland as reported also for cows [15]; therefore, the additional administration seems to significantly decrease SCC values in the successive days in milk and take part in the significant difference observed on overall SCC mean values. Moreover, the decrease of *S. aureus’* negative effects seems to be also confirmed by the lower prevalence of mastitis recorded from 30 to 90 DIM in VG2 than N-VG2.

## Conclusions

The current study represents the first investigation evaluating direct effects of vaccination against *S. aureus* in dairy Mediterranean Buffalo. Even though this study included only a small number of animals, the use of inactivated vaccine by means of the protocol B (based on label use) shows encouraging results because associated with significant lower prevalence and incidence of mastitis as well as lower SCC values, than the protocol A (based on two doses). Nevertheless, the lack of large-scale effects against IMI highlights that vaccination in MB, as in cow, may be considered only an additional tool in the control of *S. aureus* infections on farms and that its use should be always associated with excellent farm management practices to successfully improve the infections control within the herd. The assessment of its effects on the entire dairy MB herd under field conditions, as well as the in-depth analysis of the infectious dynamics, warrant further scientific attention with the goal to fully understand the potentialities of this inactivated vaccine in dairy buffalo management.

## Abbreviations

BMC: Bacteriological milk culture
Cell: Cellules
CM: Clinical mastitis
CMT: California mastitis test
DIM: Days in milk
*E. coli*: *Escherichia coli*
h: Hour
IMI: Intramammary infection
MB: Mediterranean buffaloes
mL: Milliliter
N-VG: Not-vaccinated group
SAAC: Slime-associated antigenic complex
SCC: Somatic cell count
SCM: Subclinical mastitis
*S. aureus*: *Staphylococcus aureus*
VG: Vaccinated group
